# Metal–ligand multiple bonds as frustrated Lewis pairs for C–H functionalization

**DOI:** 10.3762/bjoc.8.177

**Published:** 2012-09-18

**Authors:** Matthew T Whited

**Affiliations:** 1 Department of Chemistry, Carleton College, Northfield, MN, United States

**Keywords:** ambiphilic reactivity, C–H activation, C–H functionalization, frustrated Lewis pair, metal carbene, multiple bond

## Abstract

The concept of frustrated Lewis pairs (FLPs) has received considerable attention of late, and numerous reports have demonstrated the power of non- or weakly interacting Lewis acid–base pairs for the cooperative activation of small molecules. Although most studies have focused on the use of organic or main-group FLPs that utilize steric encumbrance to prevent adduct formation, a related strategy can be envisioned for both organic and inorganic complexes, in which "electronic frustration" engenders reactivity consistent with both nucleophilic (basic) and electrophilic (acidic) character. Here we propose that such a description is consistent with the behavior of many coordinatively unsaturated transition-metal species featuring metal–ligand multiple bonds, and we further demonstrate that the resultant reactivity may be a powerful tool for the functionalization of C–H and E–H bonds.

## Introduction

Orbital cooperation has long been recognized as an important contributor to the diverse reactivities exhibited by transition-metal systems with small-molecule substrates. The Dewar–Chatt–Duncanson model provides a paradigm for this sort of interaction, where molecules such as H_2_ and alkenes are activated by a combination of ligand-to-metal σ donation and metal-to-ligand π backbonding [1–2]. The traditional line of thought was that main-group molecules could not mimic this sort of behavior due to their more limited redox flexibility and propensity to form inert Lewis acid–base adducts, but recent work by Power, Bertrand, and others has shown that a number of unsaturated main-group compounds can exhibit electronic properties and reactivity reminiscent of transition metals [3].

A different approach was pioneered by Stephan, who demonstrated that appropriately encumbered (i.e., "frustrated") Lewis acids and bases could achieve synergistic heterolytic cleavage of H_2_ [4–6], and subsequent work in many laboratories has shown that such frustrated Lewis pairs (FLPs) may react with a variety of substrates. Most FLPs involve only main-group acids and bases (with trialkylphosphines and fluorinated triarylboranes being most common), though recent reports have extended the approach to include transition metals as Lewis acids and bases [7–8].

In this review, we show that the FLP concept may be extended to encompass certain metal–ligand multiply bonded species, provided that the metal retains an open coordination site to facilitate cooperative reactivity. Such complexes may activate various substrates through the combined action of filled and empty orbitals on adjacent atoms: a hybrid of the classical Dewar–Chatt–Duncanson paradigm and normal FLP reactivity [9–10]. M═E FLPs include two limiting scenarios: (1) early, electropositive transition metals in high oxidation states that are attached to π-basic ligands (i.e., M^δ+^═E^δ−^), and (2) late transition metals in low oxidation states attached to π-acidic ligands (i.e., M^δ−^═E^δ+^). The reactivity engendered by such a bonding situation can in some cases be quite useful in C–H functionalization schemes that require cooperative activation of substrates. One well-defined case with iridium(I) carbenes generated by multiple C−H activations is explored as a proof of principle.

Note that the purpose of this review is not to provide an exhaustive list of examples of reactivity consistent with the description of certain metal–ligand multiple bonds as FLPs, thus there will necessarily be a number of omissions. Instead, this article is presented in order to show the similarity between many M═E species and main-group FLPs and provide some inspiration for how such multiply bonded complexes may be used in C–H functionalization schemes.

## Review

### Metal–ligand multiple bonds as FLPs

#### Electronic basis for FLP behavior of metal–ligand multiple bonds

**Scheme 1:**
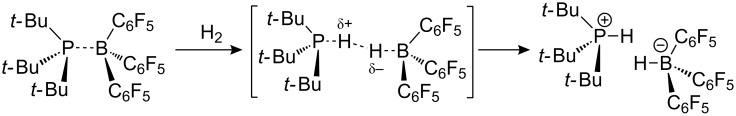
Heterolytic cleavage of H_2_ by a phosphine/borane FLP by H_2_ polarization in the P–B cavity [5,11].

**Scheme 2:**
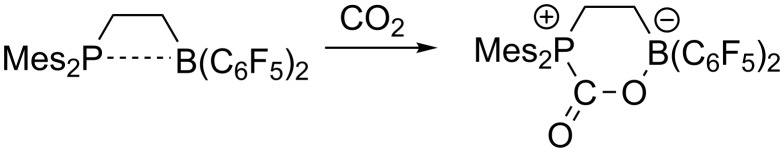
Insertion of carbon dioxide into a phosphine/borane FLP [14].

As mentioned above, most FLPs rely on steric encumbrance to minimize the interaction between an electron-rich Lewis base and an electron-poor Lewis acid. The weakly interacting acid–base pair is then capable of activating various substrates by synergistically polarizing bonds, often in a concerted fashion [6,11–14]. The reaction is favorable, because the small-molecule substrates facilitate a shift in electron density away from the electron-rich Lewis base and toward the electron-deficient Lewis acid. In the case where molecular hydrogen interacts with a phosphine/borane FLP, this occurs by the formation of a phosphonium/borate ion pair (Scheme 1). For unsaturated substrates, the reaction is better described as an insertion or cycloaddition (see Scheme 2 for a representative example), but the outcome is quite similar.

**Figure 1:**
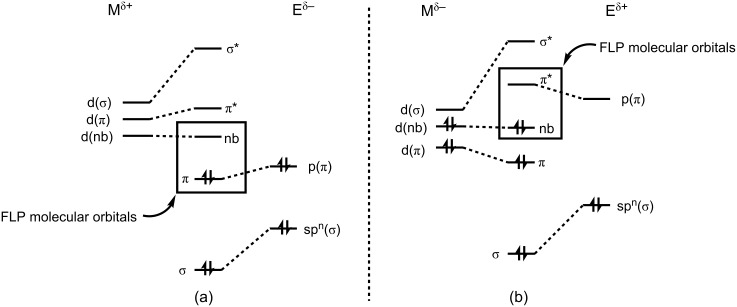
Simplified frontier-molecular-orbital diagrams for (a) M^δ+^═E^δ−^ and (b) M^δ−^═E^δ+^ FLPs (*n* = 1 for linear or terminally bound species, e.g., nitride, carbyne, linear imide, oxo, or borylene; *n* = 2 for bent or trigonal species, e.g., carbene, silylene, bent imide, amide, or boryl).

The FLP description may easily be extended to transition-metal species containing multiple bonds to ligands, provided that two conditions are met: (1) The metal must retain a vacant coordination site or be able to dissociate a ligand to provide such a site, and (2) there must be sufficient M^δ+^═E^δ−^ or M^δ−^═E^δ+^ character (typically associated with incomplete E→M or M→E π donation) to induce reactivity with polar or polarizable substrates. The first requirement is fairly straightforward: if a transition metal is coordinatively saturated, it will be unable to react as a Lewis acid or base, irrespective or how electron poor or rich it is. The second requirement can perhaps be better conveyed by using molecular-orbital diagrams. For the M^δ+^═E^δ−^ case, which we may associate with early, electropositive transition metals in their highest oxidation states, ligand-to-metal π donation is not strong enough to fully attenuate either the π basicity of the ligand or the acidity of the metal (Figure 1a). This bonding scenario is frequently encountered, for example, with the classic Group 4 imido complexes of Bergman and Wolzcanski, or the Group 5 alkylidenes of Schrock [15–16]. The reverse M^δ−^═E^δ+^ case, in which π backbonding from an electron-rich metal into a relatively electropositive ligand is insufficient to fully attenuate the basicity of the metal and/or the π acidity of the ligand (Figure 1b), is encountered for low-oxidation-state late-metal silylene [17], carbene [18], and borylene complexes [19], among others [20–23]. Either bonding situation can be described as electronic frustration [24], since sterics do not play a primary role in separating acidic and basic reactive sites on a molecule.

**Figure 2:**
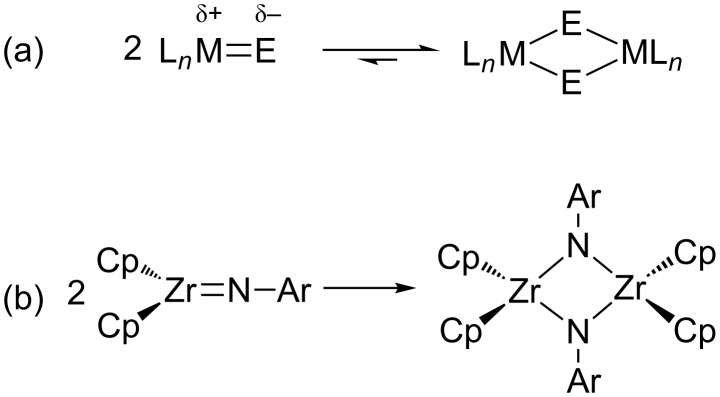
Quenching of M═E FLPs by dimerization: (a) generic M^δ+^═E^δ−^ case, and (b) Bergman's arylimido zirconium(IV) [25].

Steric effects nevertheless play an important role, as one can envision a bimolecular pathway to acid–base adduct formation (Figure 2a). Such dimerization does occur in cases with insufficient steric encumbrance (e.g., the bis-*μ*-imido zirconium complexes of Bergman, Figure 2b) [25]. Thus, as for the main-group FLPs of Stephan and others, moderately-to-severely bulky ligands must be employed to favor the most reactive monomeric M═E FLPs.

#### M═E FLPs with π-basic ligands: Reactions with unsaturated bonds

Species containing M^δ+^═E^δ−^ moieties have been known for some time, with the clearest examples being terminal imido and alkylidene complexes of early metals in their highest oxidation states. Such complexes may have substantial nucleophilic character at the multiply bonded group E [15–16,26], leading to well-defined reactions with various electrophiles as well as polarized and polarizable substrates. In many cases, these reactions resemble those explored more recently for main-group FLPs.

**Scheme 3:**
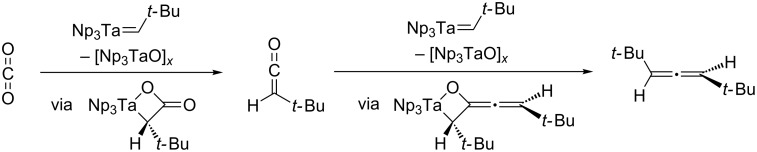
Oxygen-atom extrusion from CO_2_ by a Ta(V) neopentylidene [27].

The first type of reaction exhibited by M═E FLPs containing π-basic ligands is with polar multiple bonds such as carbonyls. The nucleophilic multiply bonded group can attack the electrophilic carbon atom, ultimately leading to metallacycle formation and frequently atom or group transfer. This type of reactivity is observed upon exposure of Schrock's tris(neopentyl)neopentylidene tantalum(V) complex to CO_2_, upon which *tert*-butylketene and then di*-tert*-butylallene are formed by consecutive oxygen-atom abstractions via metallacyclic intermediates (Scheme 3) [27].

**Scheme 4:**
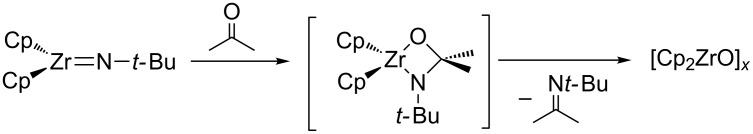
Oxygen-atom transfer from acetone at a Zr(IV) imide [28].

Similar reactions have been observed for nucleophilic imido complexes, in which imines can be formed by an oxo/imide metathesis at zirconium(IV). As with the Schrock neopentylidene, the reaction proceeds through a four-membered metallacycle, which eliminates the organic product through a [2 + 2] cycloreversion (Scheme 4) [28]. Other early metal imides may demonstrate similar reactivity, as seen in a reaction reported by Schrock for a tantalum(V) imide [29].

The reactions described above represent only a few of the many metal–ligand cooperative reactions of nucleophilic, multiply bonded species with polar multiple bonds. Related reactions have been observed for terminal oxo, sulfido, phosphinidene, and alkylidyne complexes of early transition metals (see references [30–33] for representative examples). Similar reactions can also occur in [3 + 2] fashion with azides [34].

**Scheme 5:**
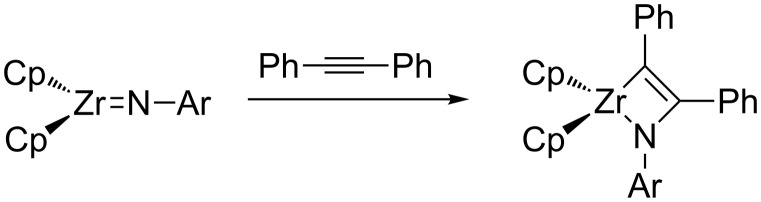
Alkyne cycloaddition at a Zr(IV) imide [38].

**Scheme 6:**
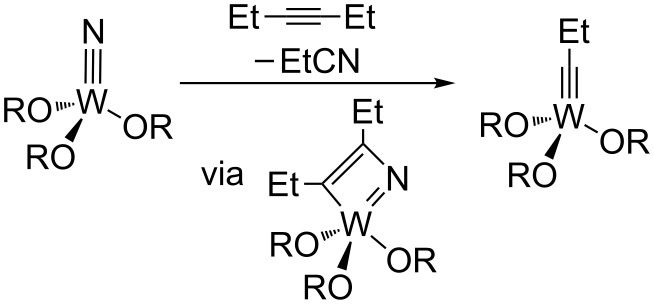
Nitrile-alkyne cross metathesis at a W(VI) nitride [40–41].

As for main-group FLPs [35], M^δ+^═E^δ−^ FLPs may also react with nonpolar unsaturated substrates, such as alkenes or alkynes, by polarizing the substrate to facilitate cycloaddition. [2 + 2] cycloadditions of M^δ+^═E^δ−^ FLPs with alkenes/alkynes have been thoroughly explored in the context of olefin metathesis (where E = CR_2_) and related variants such as alkyne and enyne metathesis [36–37]. Related reactivity is prevalent for other M^δ+^═E^δ−^ species such as imides and nitrides. Bergman's bis(cyclopentadienyl)zirconium(IV) imides, described above, will add alkenes and alkynes in [2 + 2] fashion across the Zr═NR bond (Scheme 5) [38]. This reaction is important for the hydroamination of alkynes by Cp_2_ZrX_2_ complexes, which proceeds through zirconium imido intermediates [39]. A similar [2 + 2] cycloaddition of symmetrical alkynes across a tungsten nitride is the initial step in Johnson's nitrile-alkyne cross metathesis reaction (Scheme 6) [40–41].

#### M═E FLPs with π-basic ligands: Reactions with saturated bonds

M^δ+^═E^δ−^ FLPs of the type described above have also been shown to react with a number of saturated bonds. Although it should be no surprise that such basic units would deprotonate relatively acidic N–H, O–H, and related bonds, their potential utility lies in the fact that they can also react with unpolarized H–H and C–H bonds (including those of methane). The result is a 1,2-addition of X–H across the M═E bond to give a M(X)(EH) species, which may in some cases react further.

**Scheme 7:**
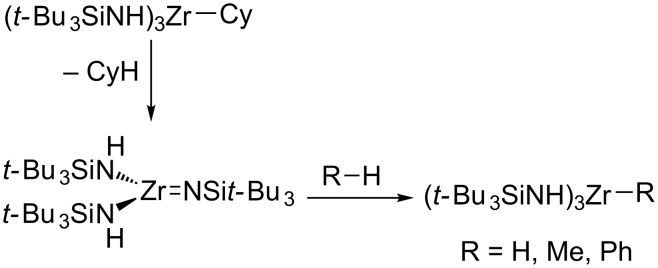
C–H and H–H addition across a zirconium(IV) imide [42].

A prominent example was reported by Wolczanski, in which a Zr(IV) silylimide can react with the C–H bonds in benzene and even methane (Scheme 7) [42]. The reaction proceeds in a manner similar to the reactions of main-group FLPs with H_2_,in which the substrate is polarized in the presence of the frustrated pair and ultimately added across it [43]. Intramolecular addition of a benzylic C–H bond across a Zr(IV) phosphinidene has been reported by Stephan [32]. Several C–H cleavage reactions have also been reported across alkylidenes and alkylidynes [44–46], and these may be viewed as the microscopic reverse of the α-hydrogen eliminations frequently utilized to generate such multiply bonded units.

The limitations of these sorts of reactions in terms of potential catalytic applications are largely related to the reluctance of the metal center to undergo redox chemistry (e.g., N–C reductive elimination to generate an amine). The systems are constructed to favor high oxidation states, so reductive elimination is quite unfavorable relative to other non-redox processes, and insertion of unsaturated bonds is generally not observed. In a sense, this limitation is similar to what is encountered in attempts to use σ-bond metathesis processes in catalysis, in which only a few specialized systems have been reported to accomplish catalytic C–H functionalization [47–49]. In fact, the bond-breaking process across metal–ligand multiple bonds is closely related to σ-bond metathesis [43], highlighting the potential of M^δ+^═E^δ−^ FLPs to activate some of the most challenging substrates.

One phenomenally useful example of heterolytic H–H cleavage across a ruthenium–amide bond, somewhat related to those described above, is found in Noyori's ruthenium hydrogenation catalysts, which utilize metal-ligand bifunctional pathways both for breaking the H–H bond and then for transferring H_2_ to polar multiple bonds [50–51]. Though the Ru–N bond polarization is not nearly as dramatic for the Noyori systems as it is for the early metal complexes described above, the nitrogen basicity and ruthenium acidity clearly play important roles in guiding the observed reactivity.

#### M═E FLPs with π-acidic ligands

The reverse situation with respect to the metal-based FLPs described above is one in which a coordinatively unsaturated metal acts as a Lewis base and a relatively electropositive π-acidic ligand acts as a Lewis acid. We might expect this situation to be less common since metals are typically formulated as cations and are more electropositive than the majority of elements normally attached to them. However, the phenomenon of metal basicity is well known [52–53], particularly for the late transition metals in low oxidation states. There are numerous cases in which M═E π bonding is inadequate to quench the electrophilicity of the multiply bonded group (particularly for heavier main-group elements, but also for carbon- and boron-based groups), affording a bonding situation that can be described as a M^δ−^═E^δ+^ FLP.

**Scheme 8:**
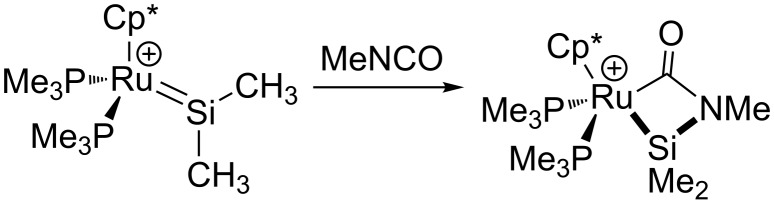
Formal [2 + 2] cycloaddition of methyl isocyanate at a ruthenium silylene [58].

Late metal silylenes, such as those explored by Tilley, often have substantial positive character at the silicon site (especially in cationic complexes), leading to reactivity that is dominated by the electrophilicity of silicon, with the metal playing a secondary role [17]. Prominent examples include the formation of base-stabilized silylenes [54–55], insertion of olefins into hydrosilylenes [56], and bimolecular redistribution of thiolates between ruthenium silyl and silylene complexes [57]. Reactivity that involves metal-ligand cooperation (in the sense described in this article) has been reported in the formal [2 + 2] cycloaddition of isocyanates to ruthenium(II) silylenes [58] (Scheme 8). These complexes do not react with nonpolar substrates (although a possible cycloaddition with azobenzene was reported), and the overall cycloaddition was found to proceed through initial nucleophilic attack at an electrophilic silylene, indicating that the metal center is not itself very reactive. However, the ability to stabilize the metallacycle is clearly derived from an enhanced transfer of electron density from ruthenium to silicon through an intervening heterocumulene. Unfortunately, retrocycloaddition to give silylene-group transfer and silaimine formation was not observed.

**Scheme 9:**
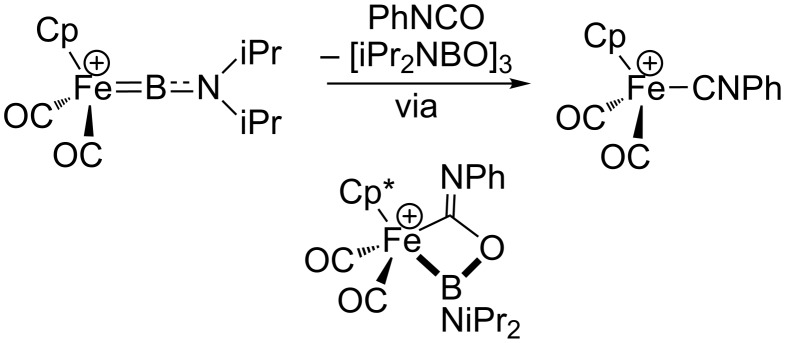
Oxygen-atom transfer from phenyl isocyanate to a cationic terminal borylene [60].

Many transition-metal borylene complexes may also be categorized as M^δ−^═E^δ+^ FLPs, undergoing reactions with heterocumulenes and other polar multiple bonds similar to those reported for silylenes [59]. The [2 + 2]-type reactions with heterocumulenes can lead to insertions or, in some cases, atom transfer [60]. One example with Aldridge's iron(II) aminoborylenes is presented in Scheme 9. In this case, Fe/B cooperation leads to scission of the C═O bond and oxygen-atom transfer to the borylene unit. As with cationic silylenes, the borylene complexes in Scheme 9 react by initial coordination of a heteroatom to the highly electrophilic boron center, followed by interaction with the metal to give a four-membered metallacycle and oxygen-atom transfer upon cycloreversion [61]. Thus, the reactions are initiated by the electrophilicity at B rather than the nucleophilic character of Fe.

**Scheme 10:**
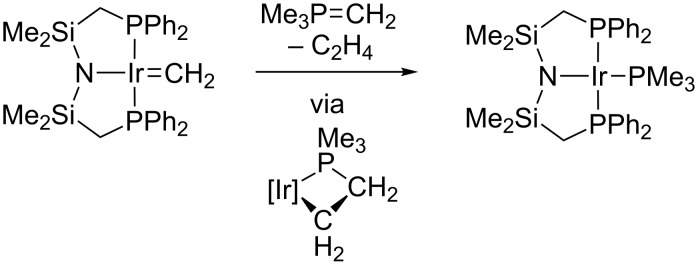
Coupling of a phosphorus ylide with an iridium methylene [62].

In contrast to the silylene and borylene examples presented above, square-planar carbene complexes of iridium(I) often react in a fashion that is dictated by the nucleophilic metal center. An early example of this type of complex was an amidophosphine-supported iridium methylene reported by Fryzuk [62–64]. With a coordinatively unsaturated and electron-rich center, this species exhibits some reactivity that is similar to the isoelectronic Vaska's complex [65], such as oxidative addition of methyl iodide. It also reacts in dipolar fashion with an in-situ-generated phosphorus ylide to release ethylene and make an iridium(I) trimethylphosphine complex (Scheme 10). More recently, Werner reported several square-planar iridium(I) carbene complexes that react with acid to selectively protonate the iridium center (i.e., the more basic/nucleophilic site) [66–67].

**Scheme 11:**
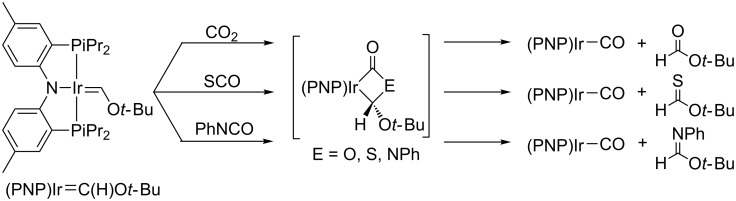
Reactions of (PNP)Ir═C(H)O*t*-Bu with oxygen-containing heterocumulenes [71].

Whited and Grubbs explored the reactivity of a related iridium(I) carbene system, supported by Ozerov's amidophosphine pincer ligand and generated by multiple C–H activations [18,68–70], with a number of heterocumulenes such as those described above. Oxygen-atom, sulfur-atom, and nitrene-group transfers to the carbene were observed when carbon dioxide, carbonyl sulfide, and isocyanates were utilized, cleanly generating the Ir(I) carbonyl as a byproduct (Scheme 11) [71].

**Scheme 12:**
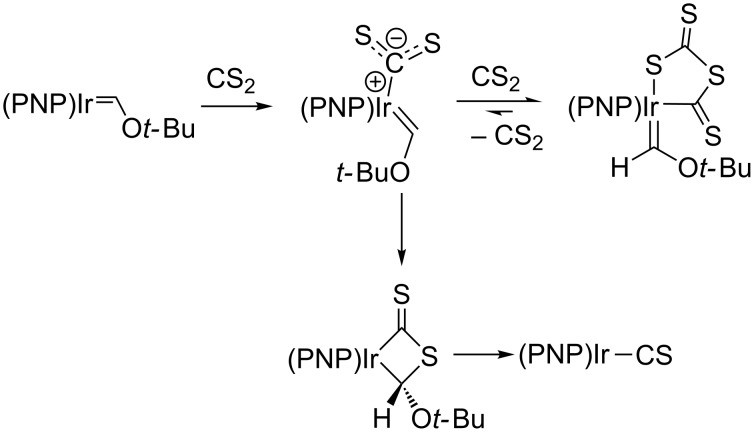
Reductive coupling of two CS_2_ units at (PNP)Ir═C(H)O*t*-Bu [73].

**Figure 3:**
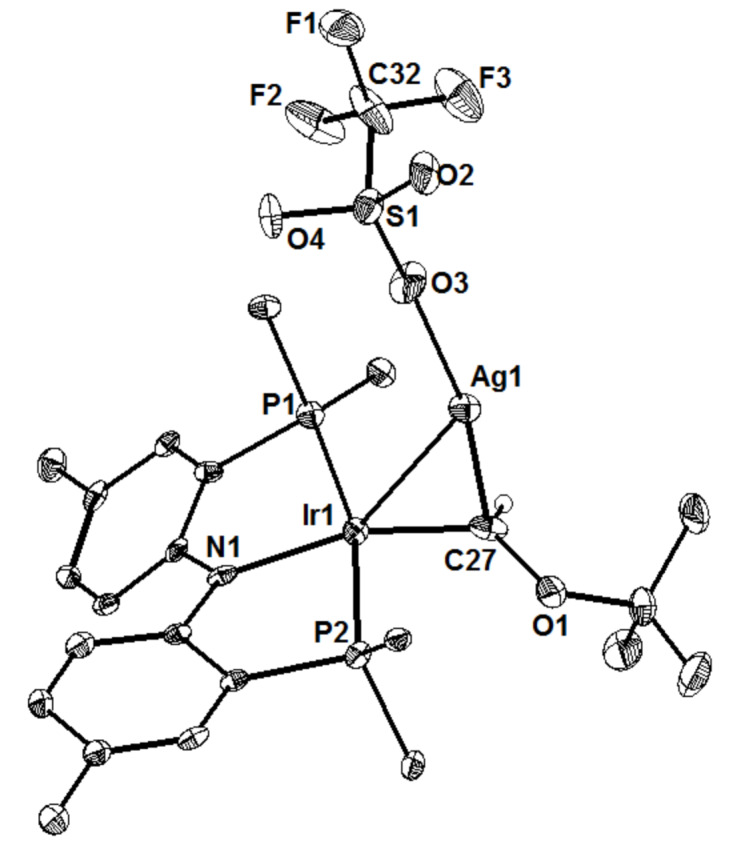
Single-crystal X-ray structure of a silver(I) triflate adduct of (PNP)Ir═C(H)O*t*-Bu with most H atoms and phosphine substituents (except *ipso* carbon atoms) omitted for clarity.

The nucleophilicity of the iridium center was demonstrated through a series of experiments. First, it was noted that the carbene complex does not react with simple nucleophiles, a departure from traditional "Fischer-type" carbene reactivity [72]. Second, the iridium center reacts with excess CS_2_ to reductively couple two carbon disulfide units, generating a metallacyclic IrC_2_S_4_^2–^ with no new bonds formed to the carbene (Scheme 12) [73]. Interestingly, this reaction is reversible and the thermodynamic product from the reaction with CS_2_ is the Ir(I) thiocarbonyl, analogous to the reactions shown in Scheme 11. Finally, though the complex does not react with simple nucleophiles, a cation–π complex is formed from the interaction of silver triflate with the Ir═C bond [74], and this complex was crystallographically characterized (Figure 3). Together, these findings showed that carbenes of this type do not exhibit traditional Fischer (electrophilic at C_α_) or Schrock-type (nucleophilic at C_α_) reactivity, and were better classified as nucleophilic-at-metal (or "Roper-type") carbenes with significant π backbonding, consistent with Roper's predicted patterns of reactivity for metal–carbon double bonds [75].

These Roper-type carbenes also reacted with organic azides and nitrous oxide via an apparent [3 + 2] cycloaddition [76–77], leading to oxygen-atom or nitrene-group transfer and formation of (PNP)Ir–N_2_ [78], and this reaction was utilized in catalytic C–H functionalization (see below). More recently, Hillhouse's nickel carbenes and imides have been shown to exhibit similar reactivity with organic azides, though reaction with CO_2_ has not been observed [77].

As described above, FLPs of the M^δ−^═E^δ+^ variety can be very useful for inducing atom or group transfer from heterocumulenes or other polar multiple bonds such as those in phosphine oxides. Compared with early-metal M^δ−^═E^δ+^ FLPs, these sorts of complexes have the advantage of greater redox activity, facilitating application in catalysis (see below). However, they also have lower reactivity due to a less polarized M═E bond, which cannot activate strong C–H or related bonds. Though H–H addition across Ir═C bonds has been reported, this almost certainly occurs by oxidative addition of H_2_ at the Ir(I) center followed by hydride migration [64,79–80]. The well-developed C–H borylation chemistry of Hartwig and others provides an indication that cooperation may be operative to some extent in the activation of C–H bonds at metal boryls [81–84], though the exact mechanism of C–H cleavage seems to depend on the nature of the metal catalyst. Nevertheless, these results do provide inspiration for the development of similar C–H functionalization catalysis involving metal carbenes or silylenes (or perhaps even electrophilic nitrenes).

### Utility of M═E FLPs in C–H functionalization

**Scheme 13:**
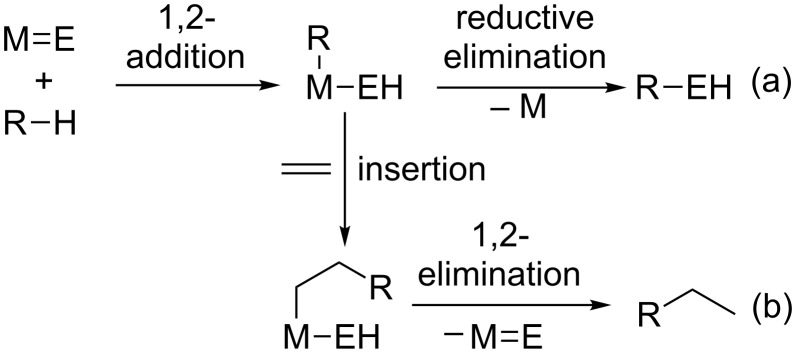
Possible routes to C–H functionalization by 1,2-addition across a polarized metal–element multiple bond.

Given the types of reactivity discussed thus far, there are several distinct routes to the functionalization of C–H (or E–H) bonds using metal–ligand multiply bonded FLPs. If C–H activation is effected by 1,2-addition across a M═E bond, then reductive elimination could result in a net C–H insertion of carbene or nitrene (Scheme 13a). This would be formally related to carbene or nitrene insertions that have been shown to occur, among other cases, at dirhodium paddlewheel complexes [85], though the specific mechanism of C–H bond breaking (and hence the reaction selectivity) would be quite different. Such a transformation has not been realized with the early metal complexes that are most reactive toward C–H bonds, probably because reductive elimination is strongly disfavored relative to the 1,2-elimination of alkane. Another possibility would be an initial 1,2-addition of a C–H bond across M═E, followed by insertion of an unsaturated substrate (olefin, alkyne) and either a 1,2-elimination (to afford a hydroalkylation or hydroarylation product) or reductive elimination as described above. However, to the best of our knowledge, this type of reactivity has not been observed at early metal imido or alkylidene complexes that can cleave C–H bonds.

**Scheme 14:**
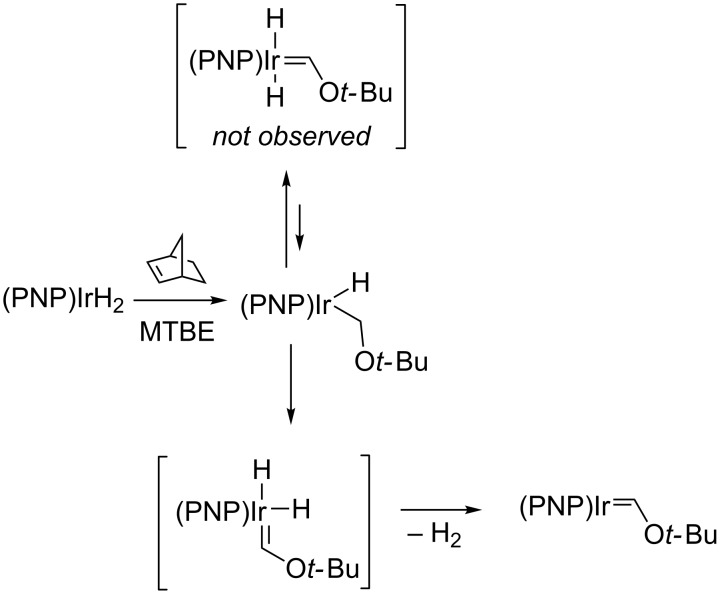
Alkoxycarbene formation by double C–H activation at (PNP)Ir [88].

An alternative route involves the generation of a M═E FLP species by multiple C−H (or E–H) activations [18,70,86]. As mentioned above, Whited and Grubbs showed that an iridium(I) carbene system that exhibited FLP reactivity could be generated by multiple C–H cleavage events at *tert*-butyl methyl ether (MTBE) (Scheme 14) [71,87]. For the (PNP)Ir system developed by Ozerov, it was found that an initial C–H activation of the most accessible methyl C–H bond in MTBE was followed by slow α-hydrogen elimination and reductive elimination of H_2_ to afford the Ir(I) alkoxycarbene. The complex could be generated stoichiometrically when norbornene was utilized as a hydrogen acceptor. The reaction was shown to be general for several methyl ethers and tetrahydrofuran, but other ethers were prone to 1,2-dehydrogenation or decarbonylation [88–89] The use of methyl amines as substrates also allowed the selective formation of dihydrido aminocarbenes, but the greater basicity of these species prevented the reductive elimination of H_2_ under any of the conditions examined [90].

**Scheme 15:**
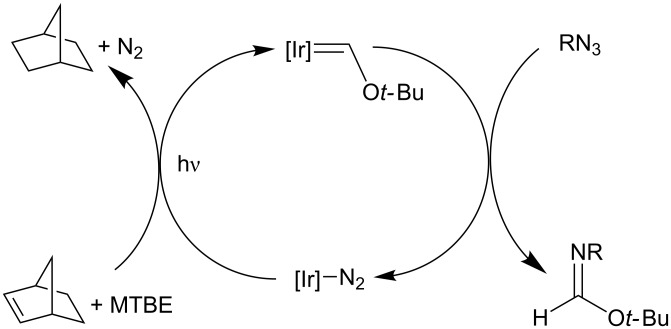
Catalytic oxidation of MTBE by multiple C–H activations and nitrene-group transfer to a M^δ−^═E^δ+^ FLP [18,78].

With this complex in hand, the coordinative unsaturation (and nucleophilicity) of the square-planar iridium center was utilized to explore a variety of atom- and group-transfer reactions, as described above. However, the iridium carbonyl, thiocarbonyl, or isocyanide products thus generated could not be incorporated into catalytic cycles due to the stability of the Ir–CO, –CS, and –CNR bonds [71,91]. A catalytic cycle was ultimately achieved following the discovery that the reaction of (PNP)Ir═C(H)O*t*-Bu with organic azides leads to the formation of *tert*-butyl formimidates and (PNP)Ir–N_2_, which is a suitable precursor for C–H activation of MTBE upon photolysis [78] (Scheme 15). Although the presence of excess azide poisoned the catalyst (presumably by irreversible formation of iridium–azide adducts), the controlled addition of azide to an MTBE solution of (PNP)Ir and norbornene, illuminated by a 23 W halogen bulb, led to efficient catalytic oxidation of MTBE. The net C–H functionalization in this case is facilitated both by the propensity of iridium to engage in multiple C–H activations to form the carbene, as well as by the M^δ−^═E^δ+^ FLP nature of the intermediate Ir(I) alkoxycarbene species.

These results not only show that species containing metal–ligand multiple bonds with the appropriate electronic structure may exhibit reactivity consistent with a FLP description, but that this reactivity may be harnessed for catalytic C–H functionalization. Several challenges remain in the catalytic cycle presented, namely that large excesses of substrate are required, and the generality of the reaction is limited by the number of substrates than can serve as carbene precursors, but these may be overcome through the design of more-selective systems for C–H activation. The reactivity observed by Grubbs also highlights the importance of hydrogen management in processes that involve generating M═E FLPs by multiple C–H activations, since the active catalysts must either be able to eliminate H_2_ without unproductive back reactions or must transfer H_2_ into a sacrificial acceptor (such as norbornene in the cycle described). All in all, these findings provide a framework both for the discovery of new reactions involving M═E FLPs and for their implementation in catalytic transformations for the functionalization of C–H and E–H bonds.

## Conclusion

In this article, we have proposed that many species containing polarized metal–ligand multiple bonds and coordinatively unsaturated metal centers may be described as analogues of the recently developed frustrated Lewis pairs involving main-group Lewis acids and bases. Although the manner in which "frustration" occurs is somewhat different (i.e., it is primarily electronic and not steric in origin), the types of reactivity observed are remarkably similar. One example in which this behavior has been used for the catalytic functionalization of C–H bonds has been elaborated, and several strategies for future utilization of such electronically frustrated species have been presented.

## Supporting Information

File 1 The single-crystal X-ray structure of [(PNP)Ir═C(H)O*t-*Bu][AgOTf] is supplied in CIF format and has been deposited with the CCDC, No. 889634.
